# Numerical simulation and experimental research on the oil removal efficiency during the oily wastewater separation by hydrocyclone

**DOI:** 10.1038/s41598-025-31480-6

**Published:** 2025-12-24

**Authors:** Zhao Shuai, Zhou Weili, Ding Laiyuan, Kong Dejun, Yu Wenwen

**Affiliations:** 1 Shandong Labor Vocational and Technical College, Jinan, 250300 China; 2 Army Engineering University, Shijiazhuang Campus, Shijiazhuang, 050003 China

**Keywords:** Oily wastewater, Oil-water separation, Hydrocyclone, Euler multiphase flow, Energy science and technology, Engineering, Mathematics and computing

## Abstract

Some oil and gas fields in China are located in the north, and in winter, oil fields face technical difficulties in separating oil and water from high viscosity condensate produced fluids. This article proposes a low-temperature oil-water separation scheme using a preheated hydrocyclone, with a focus on studying the separation effect of oil-water mixtures with different oil contents by varying the overflow diversion ratio and inlet flow rate of the hydrocyclone. The structure of the cyclone was optimized using response surface methodology (RSM) through three-dimensional modeling. The Euler multiphase flow model was used to study the distribution characteristics of the flow field and phase volume fraction of the oil-water two-phase medium inside the hydraulic cyclone under different injection parameters. The results show that when the oil content of the mixed liquid is 10% and the viscosity of the oil phase is 27-31mPa·s, an increase in the overflow diversion ratio is beneficial for the rapid separation of low-density phase media. If the diversion ratio is too high, it will cause the liquid flowing out of the overflow port to form an oily mixture again. In our experiment, the optimal overflow diversion ratio is 0.2, and the effective oil removal efficiency can reach 92%. The influence of the oil content in the mixed liquid on the oil-water separation efficiency of the cyclone is achieved through the interaction of viscous drag and centrifugal force on the radial partial pressure and tangential flow velocity. As the oil content and viscosity of the mixed liquid increase, the radial flow resistance experienced by the cylindrical and conical sections of the cyclone will increase. The radial partial pressure, pressure gradient, and radial flow velocity of the aqueous medium will also decrease, and the separation efficiency will also decrease accordingly.

## Introduction

The rapid development of China’s social economy has led to a sharp increase in energy demand. Petroleum resources, as an important component of China’s energy structure, have a significant impact on socio-economic development. The efficient development of oil fields can effectively solve the problem of insufficient oil energy supply, but the mode of water injection and displacement extraction will also generate a lot of oily wastewater with the development of oil fields. In recent years, China has generated nearly 3 billion tons of oily wastewater annually in processes such as oil extraction, refining, and petrochemicals^1,2^. If the corresponding oily wastewater cannot be effectively treated, it is easy to cause serious pollution to the ecological environment^3^. Oily wastewater mainly comes from the associated water produced in the oil and gas production process. Oil contaminated water contains various harmful and even toxic substances as well as carcinogens, such as benzene, toluene, xylene, naphthalene, styrene, etc. If discharged into rivers without manual treatment, it will cause irreversible damage to the drinking water sources and natural environment that humans rely on for survival, directly affecting human life and health^4,5^.

At present, the main treatment methods for oily wastewater in oilfield production processes include adsorption flocculation method and membrane separation method. These methods have relatively low processing efficiency but high engineering costs. Jiang et al. used the in-situ sol-gel process to synthesize titanium modified multi walled carbon nanotubes for the treatment of oily wastewater. The results showed that the transmittance of the aqueous phase could reach 89.7% when the oily wastewater was kept at room temperature for 30 min by 400 mg/L CNTs/TiO2^6^. The flocculation performance is achieved through multiple synergistic effects of charge neutralization, hydrophobic association, and adsorption bridging. Zhang et al. found that the presence of a large amount of polymers in oilfield wastewater leads to a decrease in oil-water interfacial tension, making it easier to form an emulsion system and increasing the difficulty of treatment. By using hydrophobic modified aluminum silicate sulfate as a coagulant to treat oilfield wastewater, when the content of hydrophobic modified aluminum silicate sulfate is 140 mg/L, the oil removal rate and turbidity removal rate can reach 95% and 98%, respectively^7^. In practical applications, it has been found that due to the addition of new chemicals such as heavy metals, ionic salts, and organic polymers in flocculants^8–10^, although the oil removal efficiency is high, the water quality has not been improved, and new processes are needed to treat water contaminated with heavy metals, ionic salts, and chemicals. On the other hand, the use of coagulants also increases the difficulty of secondary treatment processes, requiring the removal of coagulants from water through sedimentation, filtration, and other processes^11,12^.

Researchers have found that traditional hydrophobic membranes are prone to fouling and wetting when used to treat complex feed solutions composed of amphiphilic pollutants such as surfactants and low surface tension oils^13,14^. Yu et al. prepared alumina ceramic chip membranes and attempted to treat oily wastewater from oil fields using membrane separation method. The results showed that an increase in Al_2_O_3_ content would promote the thermodynamic instability of ceramic slurry, leading to enhanced interfacial instability^15^.Tijjani et al. studied the application of polymer membranes in the treatment of high salinity oilfield produced water, and found that the organic matter in the surface energy of oilfield produced water affects the durability and scaling tendency of polymer membranes, leading to an impact on their performance^16^.Membrane fouling and wetting may significantly reduce permeate flux and salt rejection rate, ultimately leading to membrane distillation process failure^17^.

For the rapid production of oil fields, both flocculation sedimentation process and membrane separation technology have relatively long processing cycles. The hydraulic cyclone with the function of rapid centrifugal separation based on the density difference between two phases is receiving increasing attention in the petrochemical industry. Liu and Jia et al. believe that the particle size of oil droplets is the main factor affecting the separation performance of oil-water cyclones. They have developed a cyclone structure that can achieve separation after reconstructing discrete oil droplet particle sizes, and completed numerical simulation studies on the internal flow field characteristics and oil-water separation process of the particle size reconstructed cyclone using Euler Euler method and Population Balance Model (PBM)^18,19^. Jing et al. investigated the impact of using a hydraulic cyclone for high viscosity heavy oil sand removal process in the study of heavy oil extraction, Based on algebraic slip mixture, Reynolds stress and discrete-phase models, the hydrocyclone desanding characteristics of heavy oil with the water cut were studied by computational fluid dynamics (CFD) numerical simulations^20^. The hydraulic cyclone has the advantages of simple structure, no moving parts, compact volume, high separation efficiency, and low cost^21,22^.In addition, it is also used in typical engines to remove moisture from lubricating oil, ensuring the normal use of the engine. Meanwhile, by removing the amount of water, the current state of the engine can be determined.

However, some oil fields in China are located in the north, such as Daqing Oilfield, Songyuan Oilfield, Dagang Oilfield, and Bohai Oilfield. When using hydrocyclones to treat oilfield oily wastewater in winter, the oil-water separation efficiency is greatly affected by temperature, especially for high viscosity and emulsion mixtures^23,24^. Based on the above background, a hydraulic cyclone experimental platform with stirring and heating function was independently developed and processed. The oil-water mixture is heated to 70 ℃ by a preheating mixer before flowing into the cyclone, and then transported to the hydraulic cyclone by a variable frequency pump to complete the oil-water separation process. This work mainly studies the influence of parameters such as temperature, flow rate, and injection pressure on the separation efficiency of high viscosity oily wastewater, aiming to solve the problem of rapid and efficient separation of oilfield oily wastewater under low temperature conditions.

## Experimental design and numerical simulation

### Experimental design

**Fig. 1:**
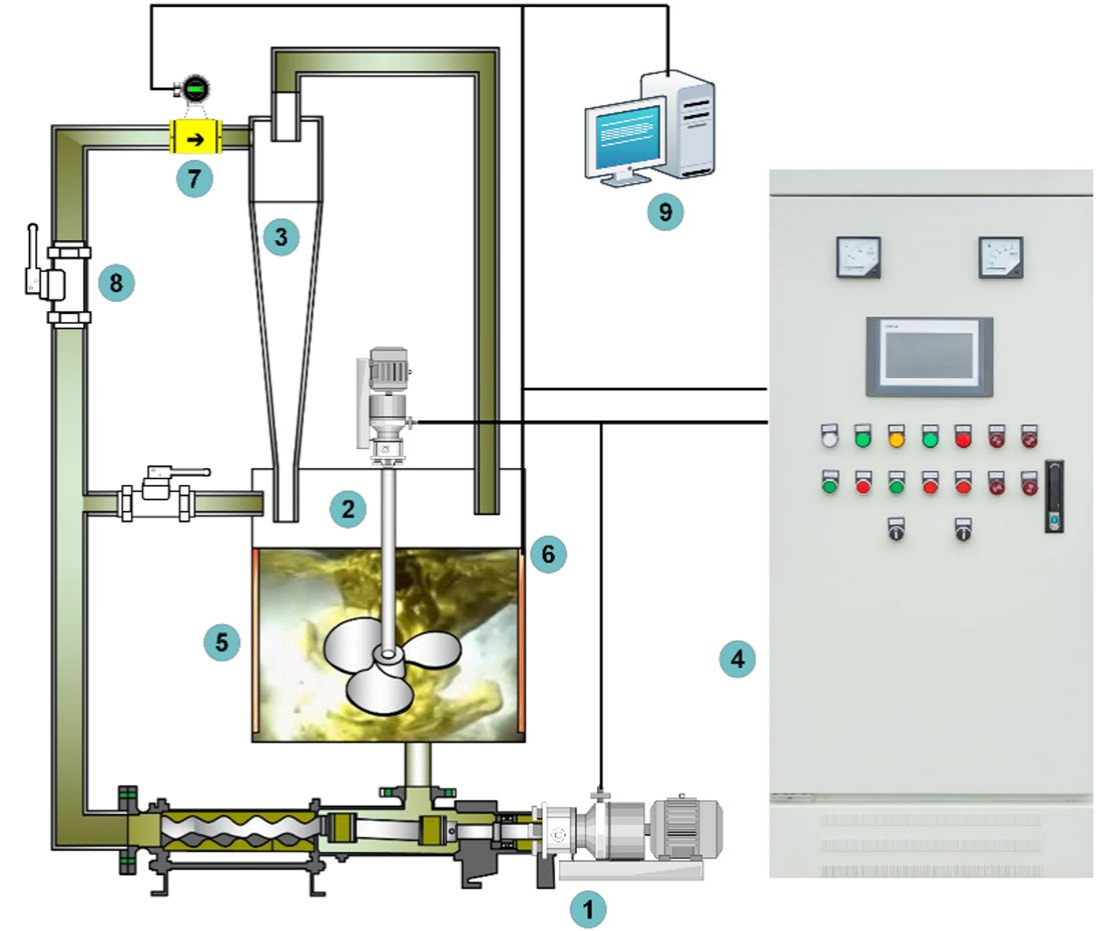
Schematic diagram of oil-water separation experimental system for hydraulic cyclone. (1) Variable frequency screw pump; (2) Preheat mixing drum; (3) Main body of hydraulic cyclone separator; (4) Power control cabinet; (5) heating rods; (6) Temperature sensors; (7) pressure sensors; (8) valves; (9) Computers

The independently built experimental platform for a hydraulic cyclone with stirring and heating functions is shown in Fig. 1. The system mainly consists of a variable frequency screw pump, a preheating mixing drum, and a hydraulic cyclone. The bottom and overflow ports of the hydraulic cyclone are designed to be detachable for easy size changes. The flow sensor is installed at the inlet of the hydraulic cyclone, and an emergency pressure relief valve is installed for the safety of the experimental process.

### Experimental materials and procedures

The oily wastewater used in the experiment came from a certain oilfield in the Bohai Sea region of China. During the construction process, the oily wastewater was randomly collected multiple times, and the oil content was measured to be 9.47–17.64%, with an oil phase density of 0.924 g/cm^3^; The viscosity of the oil phase measured at different temperatures is shown in Table 1.

**Table 1:** Characteristics of viscosity variation of oil phase in a certain oilfield with temperature.

Temperature /℃	Viscosity /mpa·s	Density /kg/m^3^
0	107 ~ 113	924
20	68 ~ 74	913
45	33 ~ 38	906
70	27 ~ 31	903

In order to facilitate the calculation of oil-water separation efficiency, the oily wastewater is reconfigured after being allowed to settle and stratify. The oil phase volume fractions are set to 10%, 20%, and 30% by extracting the lower layer water. The experiment uses a variable frequency screw pump with a pump capacity range of 0-4.5m^3^/h. By adjusting the frequency (0 ~ 50 Hz) to change the pump volume and inlet pressure, the pressure change is recorded by a pressure sensor installed at the inlet of the hydraulic cyclone. The hydraulic cyclone is processed using S304 stainless steel material, and the structural parameters are shown in Table 2.

**Table 2:** Structural parameters of Hydrocyclones.

Name	Symbol	Numerical value
Entrance cross-sectional area	A_1_	625.00 mm^2^
Overflow pipe diameter	D_1_	4 ~ 10 mm
Overflow pipe length	L_1_	25.00 mm
Depth of overflow pipe	H_1_	12.50 mm
Interval between overflow pipe and cylindrical section	L_2_	6.25 mm
Diameter of cylindrical segment	D_2_	125.00 mm
Cylinder segment height	H_2_	75.00 mm
Cone segment length	L_3_	316 mm
taper angle	α	11.75°
Bottom outlet diameter	D_3_	2 ~ 8 mm

**Fig. 2:**
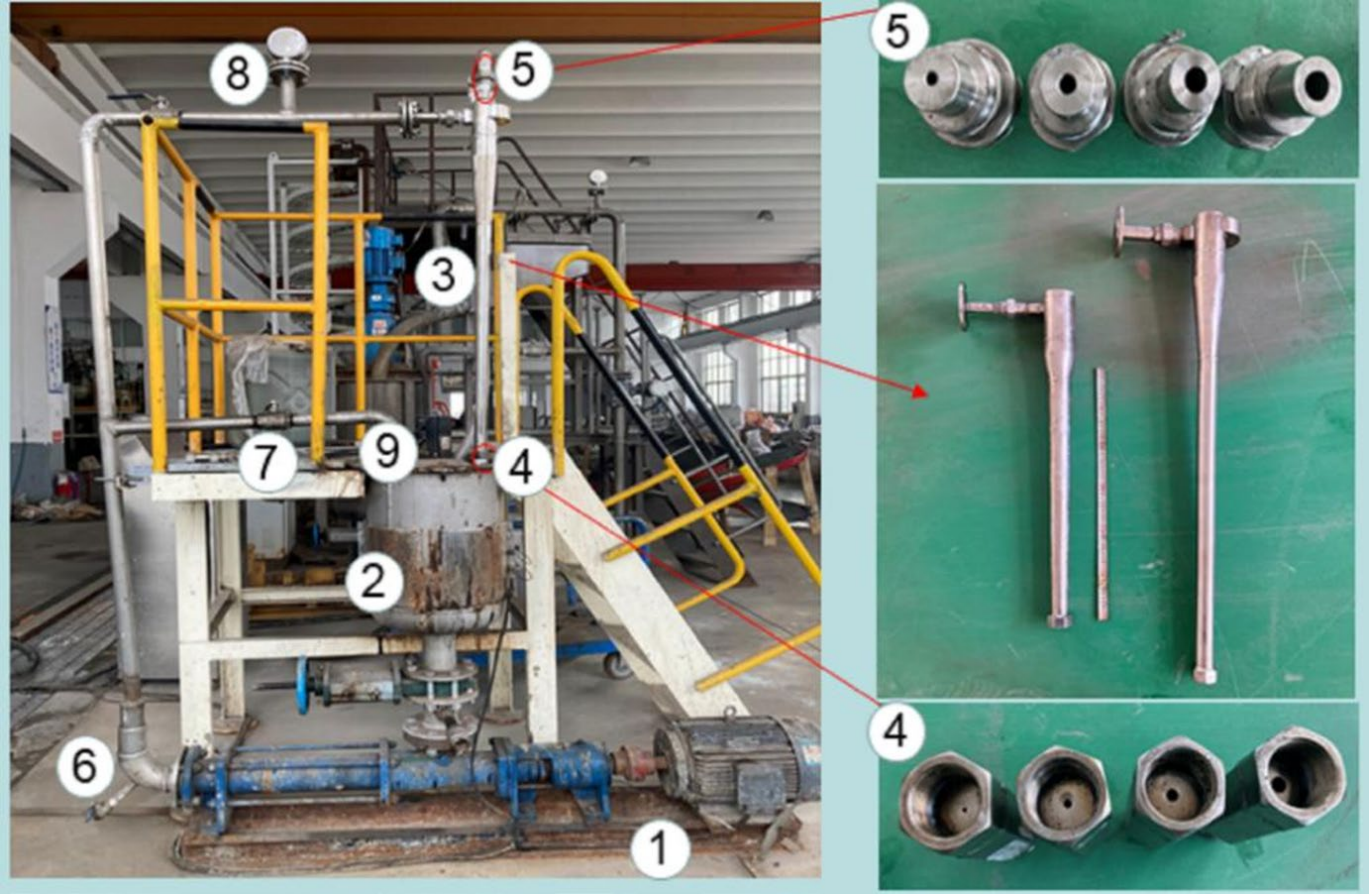
The Oil Water Separation Experimental System. (1) Variable frequency screw pump; (2) Preheat mixing drum; (3) Main body of hydraulic cyclone separator; (4) Detachable bottom outlet; 5 detachable overflow ports; 6 Emergency relief valve (normally closed); 7 pressure relief valve (normally closed); 8 flow sensors; 9 Temperature Sensor

The overflow port and bottom flow port are designed to be detachable, making it easy to replace joints with different aperture sizes, as shown in Fig. 2. The experiment was conducted in a low-temperature environment, with a laboratory temperature of 2 ℃. Before the experiment begins, place the prepared oily wastewater in a preheated mixing tank. Keep the indoor temperature constant, activate the heating function of the preheating mixing drum, and control the temperature of the mixed liquid in the drum at 70 ℃Turn on the stirring function, mix the oil-water mixture evenly, and then turn on the variable frequency screw pump. Set the pump volume to 0.5, 1.0, 1.5, 2.0, 2.5, 3.0, 3.5, 4.0, 4.5 m^3^/h by adjusting the frequency. After the cyclone stabilizes (1 min), samples are taken from the overflow and bottom flow ports respectively to calculate the oil volume fraction.

### Numerical model

**Fig. 3:**
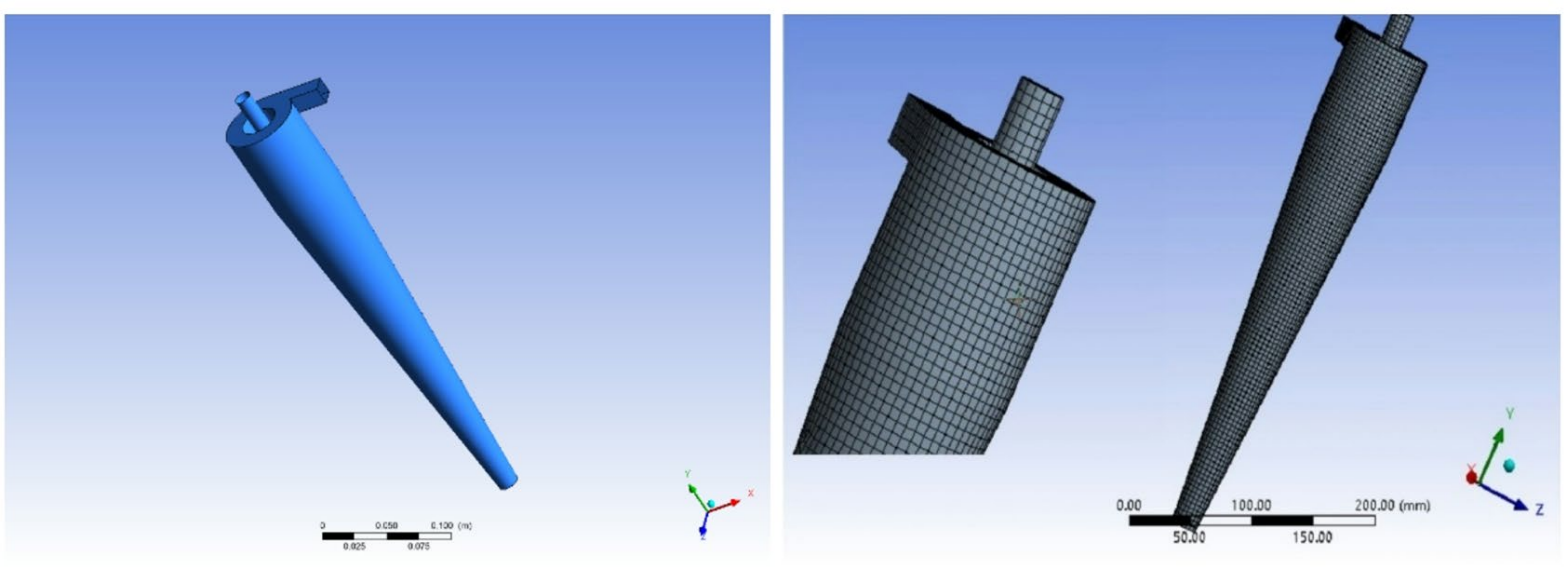
Structural Model and Grid Division of Hydrocyclone

The model selected in this article is a tangential inlet hydraulic cyclone, consisting of an inlet pipe, a cylindrical section, a conical section, an overflow pipe, and a bottom flow port. According to the structural parameters of the cyclone in Table 2, a hydraulic cyclone structure model was drawn using Inventor. Import the structural model into the Ansys Workbench module to complete hexahedral mesh partitioning and verify mesh independence based on changes in overflow pressure drop. The final selected grid size is 2.8 mm, with a total of 224,704 cell grids and 287,907 nodes; Use Fluent to simulate oil-water separation, as shown in Fig. 3.

The initial density of the main medium water phase in the numerical simulation calculation process is 1000 kg/m^3^, and the viscosity is 1.00 mPa·s. The initial density of the oil phase is 924 kg/m^3^ and the viscosity is 113 mPa·s. After heating, the density of the oil phase is 903 kg/m^3^ and the viscosity is 31mpa.s. The form of the mixed liquid entering the cyclone is set as the velocity inlet. The initial oil droplet size is randomly distributed between 50 and 800 μm. Both the overflow port and the bottom flow port are set as pressure outlets (free outflow). The wall boundary is set to non-slip and impermeable conditions. Considering the influence of turbulent anisotropy effects such as rotational flow and changes in surface curvature in the flow direction, the Reynolds stress model (RSM)^25,26^ was selected as the turbulence calculation model, and the Eulerian multiphase flow model^27^ was used as the calculation model.

The mass conservation and momentum conservation equations of the Euler multiphase flow model are as follows:

1 $$\:\partial\:/\partial\:t\left({\alpha\:}_{k}-{\rho\:}_{k}\right)+\nabla\:\cdot\:\left({\alpha\:}_{k}{\rho\:}_{k}\overrightarrow{{u}_{k}}\right)=0$$

2 $$\:\partial\:/\partial\:t\left({\alpha\:}_{k}{\rho\:}_{k}\overrightarrow{{u}_{k}}\right)+\nabla\:\left({\alpha\:}_{k}{\rho\:}_{k}\overrightarrow{{u}_{k}}\overrightarrow{{u}_{k}}\right)=-{\alpha\:}_{k}\nabla\:p+{\alpha\:}_{k}{\rho\:}_{k}\overrightarrow{g}+\nabla\:\overrightarrow{{\tau\:}_{k}}+\overrightarrow{F}$$

3 $$\:{\sum\:}_{k=1}^{n}{\alpha\:}_{k}=1$$

In the formula, t is time; α_k_ is the volume fraction of the k phase; ρ_k_ is the density of the k-phase; u_k_ is the fluid velocity; P is pressure; $\:\overrightarrow{\mathrm{g}}$ is the acceleration due to gravity; $\:\overrightarrow{{{\uptau\:}}_{k}}$ is the tensile stress;$\:\:\overrightarrow{\mathrm{F}}$ is the interphase force.

The main interaction force in the oil-water cyclone separation process is drag force,

4 $$\:\overrightarrow{F}=\frac{3}{4}\left(\frac{{C}_{D}}{d}\right){\alpha\:}_{O}{\rho\:}_{W}\left|{\rho\:}_{O}-{\rho\:}_{W}\right|{\rho\:}_{W}\left(\overrightarrow{{u}_{o}}-\overrightarrow{{u}_{W}}\right)$$

In the formula, u_o_ and u_w_ represent the radial fluid velocities of oil and water phases, respectively; d Cyclone diameter; C_D_ is the drag coefficient; ρ_o_ and ρ_w_ are the densities of oil and water phases, respectively; The subscripts o and w represent the oil phase and water phase, respectively.

In this simulation process, when describing the particle size distribution of dispersed phases in multiphase flow systems, the impact of particle size changes caused by oil droplet coalescence and fragmentation on drag force, tensile stress, and flow velocity was considered, and the Community Balance Model (PBM) was selected. Describing the equilibrium of particles by adding an equilibrium equation based on the conservation of momentum and energy^28^. Considering the influence of vortex intensity on the particle size distribution of oil droplets during the cyclone separation process, the coalescence mechanism of oil droplets is divided into viscous coalescence and inertial coalescence, which are independently calculated. The Turbulent coalescence model is selected, and the Hamaker constant is set to a default value of 2.3 × 10^− 20^. The fragmentation model is a particle fragmentation rate model (Luo) based on the theory of isotropic uniform turbulence and probability statistics. It mainly calculates the size distribution of sub particles and sets the surface tension coefficient to 0.0728 N/m.

The conservation equation of the community balance model is,

5 $$\:\partial\:/\partial\:t\left[n\left(V,t\right)\right]+\nabla\:\cdot\:\left[\overrightarrow{u}\cot\:n\left(V,t\right)\right]=S\left(V,t\right)$$

In the formula, V is the volume of the droplet, t is time, $\:\overrightarrow{u}$ is the liquid flow rate, n is the number density, and S(V, t) is the source term for droplet coalescence and fragmentation. The specific expression is as follows,

6 $$\:S\left(V,t\right)={B}_{C}\left(V,t\right)-{D}_{C}\left(V,t\right)+{B}_{B}\left(V,t\right)-{D}_{B}\left(V,t\right)$$

7 $$\:{B}_{C}\left(V,t\right)=\frac{1}{2}{\int\:}_{0}^{\infty\:}a\left(V-{V}^{{\prime\:}},{V}^{{\prime\:}}\right)n\left(V-{V}^{{\prime\:}},t\right)n\left({V}^{{\prime\:}},t\right)d{V}^{{\prime\:}}$$

8 $$\:{D}_{C}\left(V,t\right)={\int\:}_{0}^{\infty\:}a\left(V,{V}^{{\prime\:}}\right)n\left(V,t\right)n\left({V}^{{\prime\:}},t\right)d{V}^{{\prime\:}}$$

9 $$\:{B}_{B}\left(V,t\right)={\int\:}_{0}^{\infty\:}\rho\:g\left({V}^{{\prime\:}}\right)\beta\:\left(\left(V\right)|{V}^{{\prime\:}}\right)n\left({V}^{{\prime\:}},t\right)d{V}^{{\prime\:}}$$

10 $$\:{D}_{B}\left(V,t\right)=g\left(V\right)n\left(V,t\right)$$

In the formula, a is the coalescence frequency;$\:\:{B}_{C}\left(V,t\right)$ and $\:{D}_{C}\left(V,t\right)$ are the droplets generated or destroyed during the coalescence of oil phases; $\:{B}_{B}\left(V,t\right)$ and$\:{D}_{B}\left(V,t\right)$ represent the droplet generation and fragmentation caused by oil phase fragmentation, respectively.

## Results and discussion

The calculation method for oil removal efficiency is based on the ratio of the oil volume fraction at the bottom outlet to the oil volume fraction at the inlet, that is

11 $$\:\mathrm{E}=\left(1-\frac{{C}_{u}}{{C}_{i}}\right)\times\:100\%$$

Among them, Cu is the volume fraction of oil content in the bottom outlet; Ci is the volume fraction of oil content at the inlet.

### Influence of outlet diameter of cyclone on oil removal efficiency

The outlet of the hydraulic cyclone includes two positions: the overflow port and the bottom flow port. In order to verify the effect of changes in the size of the overflow and bottom flow ports on the oil removal efficiency of oily wastewater, an experiment was conducted by changing the overflow diversion ratio by replacing different sizes of overflow and bottom flow ports when the oil content of the wastewater was 10% and the temperature was 0–2 ℃. The results are shown in Table 3.

**Table 3:** Influence of diversion ratio on oil removal efficiency of oily wastewater.

Serial number	Overflow-Bottom outlet diameter/mm	Overflow diversion ratio	Oil volume fraction at the bottom outlet/%	Oil volume fraction at overflow outlet/%	Oil removal efficiency/%
1	4–14(No tailpipe)	0.1	1.70%	91.00%	83.00%
2	4–8	0.2	0.80%	83.70%	92.00%
3	4–6	0.3	1.20%	64.70%	88.00%
4	6–8	0.4	1.50%	66.70%	85.00%
5	6–6	0.5	1.10%	59.40%	89.00%
6	8 − 6	0.6	0.80%	57.20%	92.00%
7	6 − 4	0.7	0.60%	55.30%	94.00%
8	8 − 4	0.8	0.30%	52.60%	97.00%
9	10 − 4	0.9	0.30%	47.70%	97.00%
10	10 − 2	0.95	0.20%	44.80%	98.00%

**Fig. 4:**
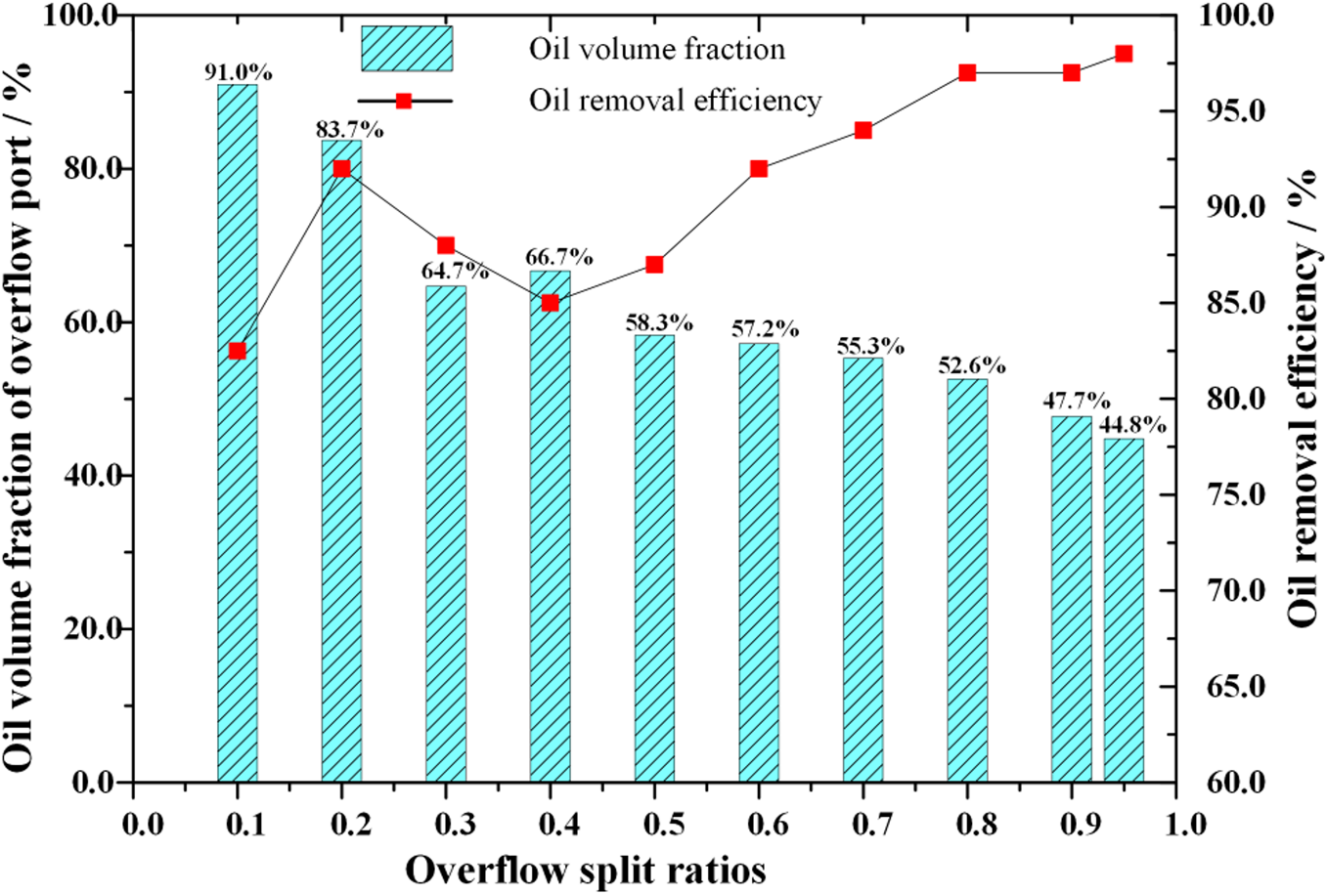
Influence of overflow diversion ratio on oil removal efficiency

As the overflow diversion ratio increases, the oil volume fraction at the bottom outlet exhibits segmented characteristics When the overflow diversion ratio increases from 0.1 to 0.2, the oil volume fraction at the bottom outlet decreases from 1.7% to 0.8% When the overflow diversion ratio continues to increase to 0.4, the oil volume fraction at the bottom outlet also shows an increasing trend, gradually increasing to 1.5% After the overflow diversion ratio exceeds 0.4, the oil volume fraction at the bottom outlet shows a decreasing trend When the overflow diversion ratio continues to increase to 0.95, the oil volume fraction at the bottom outlet drops to 0.2%. When the overflow diversion ratio increases from 0.1 to 0.95, the overall oil volume fraction at the overflow outlet shows a decreasing trend, from 91% to 44.8%. As shown in Fig. 4, the variation trend of oil removal efficiency and oil volume fraction at the overflow port with the overflow diversion ratio. During the process of increasing the overflow diversion ratio from 0.1 to 0.95, the oil removal efficiency of the mixed liquid gradually increased from 83% to 98%, but the oil volume fraction at the overflow port decreased from 91% to 44.8%.

**Fig. 5:**
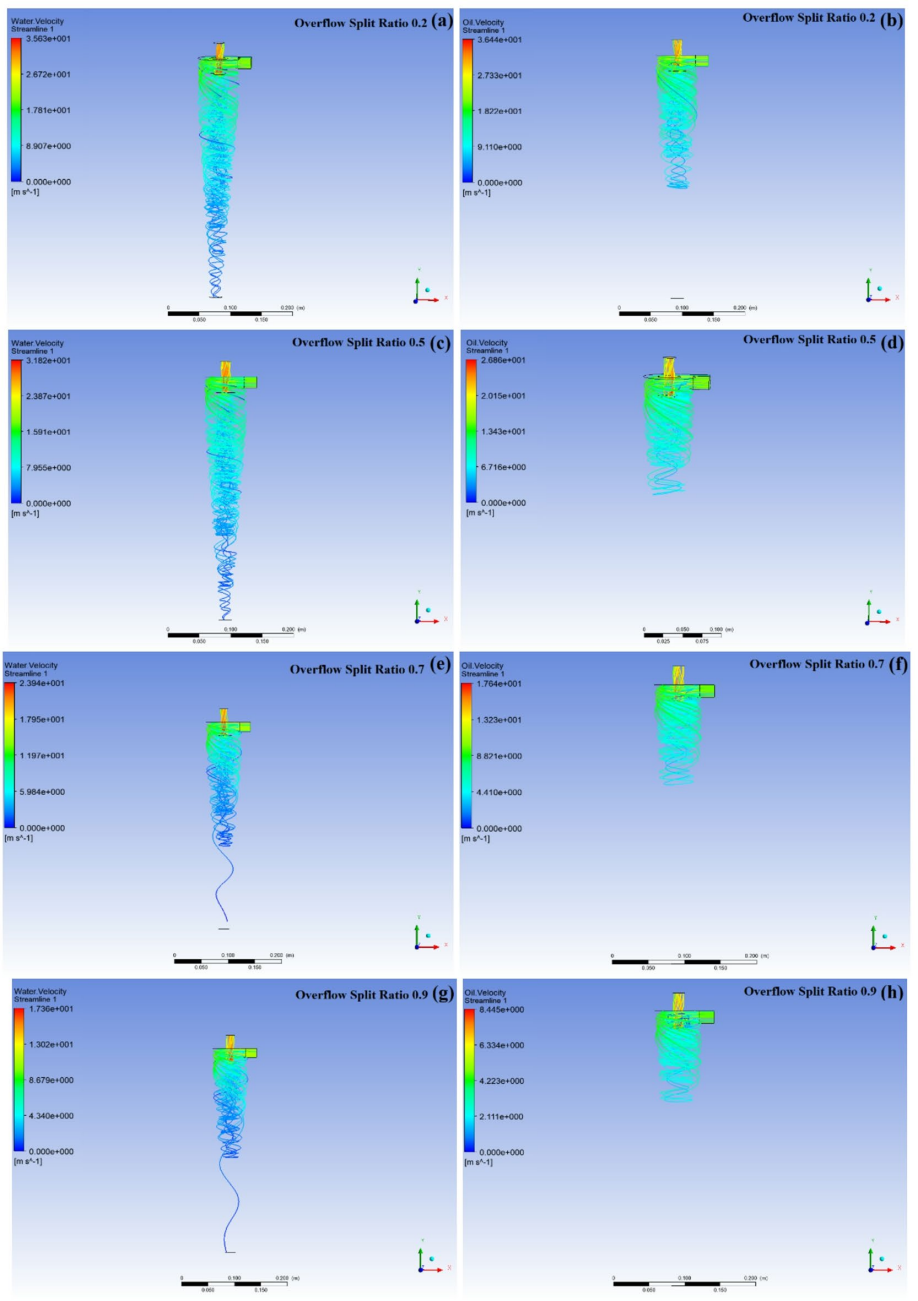
Influence of overflow diversion ratio on the internal flow field of the hydraulic cyclone

Combining the changing characteristics of the internal flow field of the hydraulic cyclone, as shown in Fig. 5. As the overflow diversion ratio increases, there are differences in the flow field distribution characteristics of the mixed liquid inside the hydraulic cyclone. When the overflow diversion ratio is lower than 0.5, the centrifugal force field inside the hydraulic cyclone is strong, and the denser water phase is thrown towards the wall of the hydraulic cyclone due to centrifugal action and moves along the outer layer of the hydraulic cyclone to the bottom outlet for discharge, as shown in Fig. 5 (a) and (c). But as the overflow diversion ratio increases to 0.7, the flow field of the aqueous medium also changes. As the diversion ratio increases, the turbulence level in the forced vortex zone increases, and the water phase still tends to move towards the wall due to centrifugal effect, but some water phases mix into the overflow port due to the enhanced internal swirl. This is because increasing the diversion ratio will weaken the strength of the outer vortex, while enhancing the carrying effect of the inner vortex on the water phase, reducing the efficiency of transporting the water phase to the bottom flow port, as shown in Fig. 5e and g. For oil phase media, as shown in Fig. 5b, d, f, and h, an increase in the overflow diversion ratio leads to an increase in the inner layer swirl intensity, and all oil phase media flows out from the overflow port. And the effective height occupied by the oil phase medium inside the hydraulic cyclone chamber is reduced, which helps to improve the size of the hydraulic cyclone chamber.

**Fig. 6:**
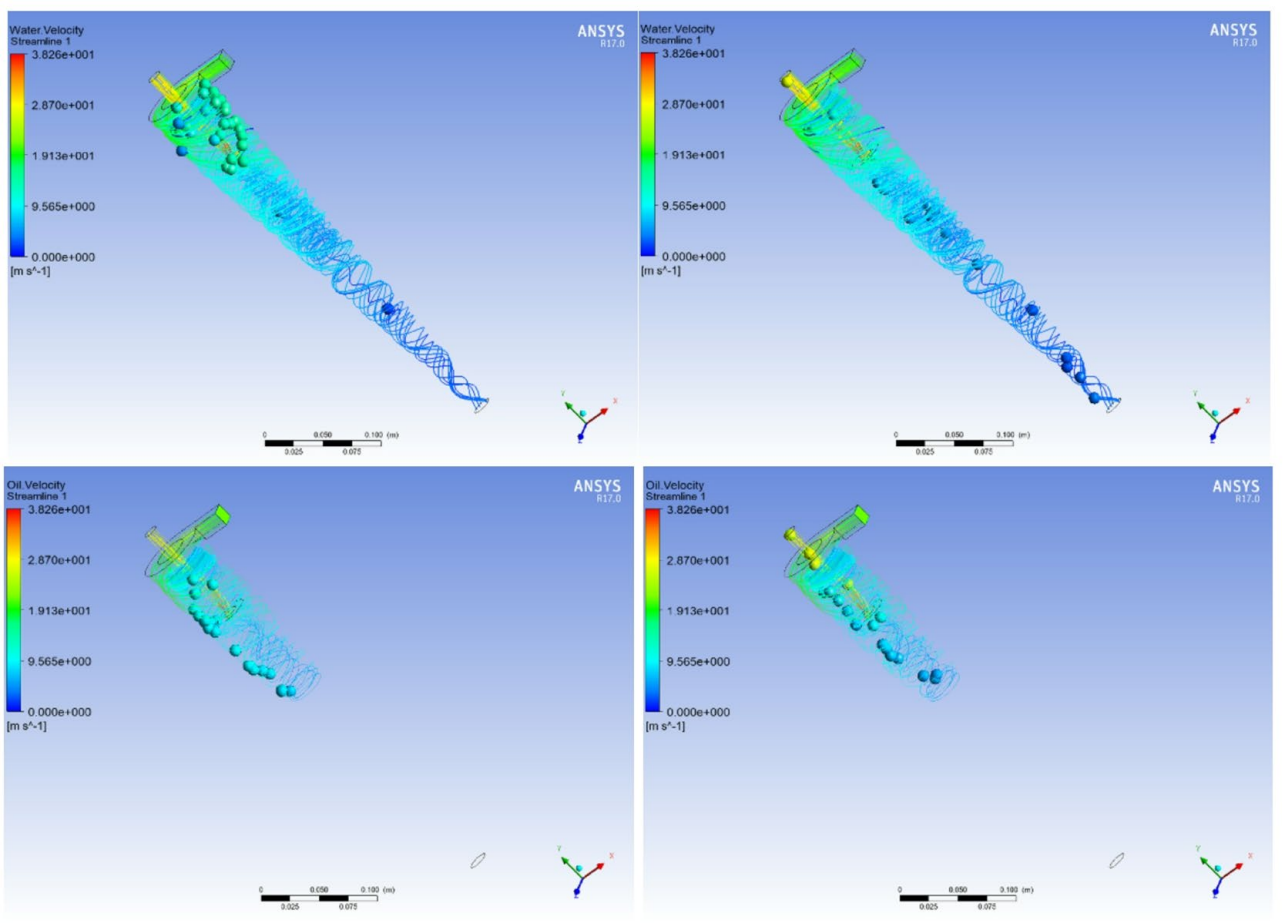
Flow field tracing of water and oil when overflow diversion ratio is 0.2

During the process of increasing the overflow diversion ratio, the space for the oil phase medium to undergo swirling in the cylindrical and large cone sections of the hydraulic cyclone decreases. This indicates that an increase in the overflow diversion ratio is beneficial for the rapid separation of low-density phase media. Combined with the streamline distribution density of the aqueous medium, an increase in the overflow diversion ratio also leads to an increase in the volume fraction of the aqueous medium flowing out from the overflow port and a decrease in the volume fraction flowing out from the bottom flow port, indicating that the liquid flowing out of the overflow port forms an oily mixture again. When the oil content of sewage is 10%, the changes in oil volume fraction at the bottom outlet, oil volume fraction at the overflow outlet, and oil removal efficiency are comprehensively considered. The optimal overflow diversion ratio of 0.2 was ultimately selected to complete the optimization experiment of process parameters for the hydraulic cyclone. At this time, the diameter of the overflow port is 4 mm, and the diameter of the bottom flow port is 8 mm. The oil phase medium in the mixed liquid mainly flows out from the overflow port, while the water phase medium mainly flows out from the bottom flow port, as shown in Fig. 6. The effective oil removal efficiency can reach 92%.

### Influence of Inlet flow rate on oil removal efficiency

**Fig. 7:**
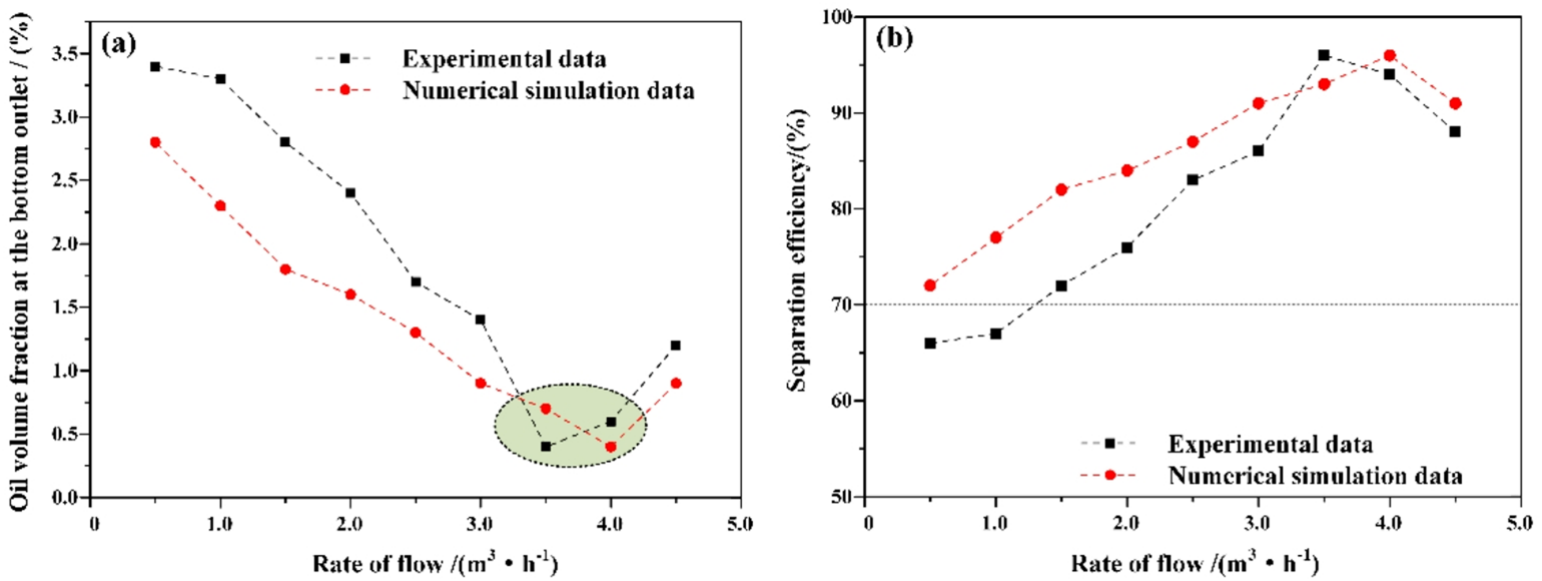
Separation effect of hydraulic cyclone (**a**) Oil volume fraction at the bottom outlet (**b**) Separation efficiency

On the basis of selecting a hydraulic cyclone overflow diversion ratio of 0.2, numerical simulations and experiments were conducted to investigate the effect of inlet flow velocity on the oil removal efficiency of the mixed liquid at an oil content of 10%. The results are shown in Fig. 7.

With different inlet flow rates, the oil volume fraction at the bottom outlet of the hydraulic cyclone exhibits a segmented distribution characteristic, as shown in Table 4. When the inlet flow rate increases from 0.5 m^3^/min to 4.0 m^3^/min, with the increase of inlet flow rate, the oil volume fraction at the bottom outlet gradually decreases from 2.8% to 0.4%, and the effective oil removal efficiency increases from 72.0% to 96.0%. When the inlet flow rate exceeds 4.0 m^3^/min, the oil volume fraction at the bottom outlet gradually increases to 0.9%, and the effective oil removal efficiency also decreases to 91.0%.

**Table 4:** Influence of Inlet flow on oil removal efficiency of oily Wastewater.

Flow rate /(m^3^·min^− 1^)	Inlet oil content/%	Bottom outlet oil volume fraction (experimental)/%	Bottom outlet oil volume fraction (simulated)/%	Separation efficiency (experimental)/%	Separation efficiency (simulated)/%
0.5	10%	3.40%	2.80%	66.0%	72.0%
1	10%	3.30%	2.30%	67.0%	77.0%
1.5	10%	2.80%	1.80%	72.0%	82.0%
2	10%	2.40%	1.60%	76.0%	84.0%
2.5	10%	1.70%	1.30%	83.0%	87.0%
3	10%	1.40%	0.90%	86.0%	91.0%
3.5	10%	0.40%	0.70%	96.0%	93.0%
4	10%	0.60%	0.40%	94.0%	96.0%
4.5	10%	1.20%	0.90%	88.0%	91.0%

**Fig. 8:**
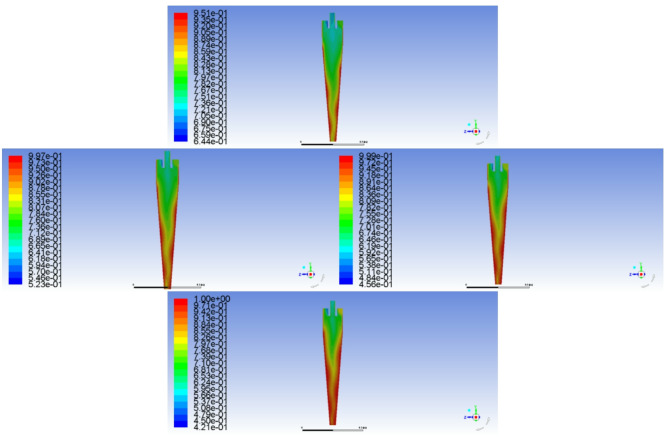
The Effect of Flow Rate Changes on the Density of Phase State Distribution in Water Phase Media

**Fig. 9:**
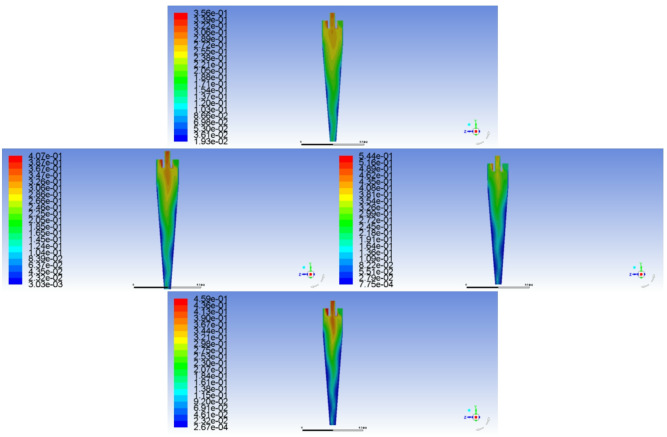
The Effect of Inlet Flow on the Density Distribution of Oil Phase Media

The influence of the inlet flow rate on the phase distribution of the mixed liquid in the hydraulic cyclone is shown in Figs. 8 and 9. Due to the effects of vortex suction, gravity, and centrifugal force, the cone section of the hydraulic cyclone is divided into a forced vortex zone at the center and a semi free vortex zone at the edge. After separation and settling in the cylindrical section, the aqueous medium descends along the cone section of the hydraulic cyclone and exhibits wall attachment characteristics in the semi free vortex zone at the edge of the hydraulic cyclone. As the inlet velocity increases, the distribution density of the aqueous medium in the bottom flow increases. When the inlet flow rate increases from 0.5 m^3^/min to 4.0 m^3^/min, the increase in inlet flow rate will enhance centrifugal force, making the oil phase more concentrated in the central area of the cyclone. The light phase, due to its low density, accumulates towards the center of the cyclone under centrifugal force to form an oil core, which is ultimately discharged from the overflow port. The heavy phase, due to its high density, is thrown towards the inner wall of the cyclone and moves in a spiral motion along the wall, and is discharged from the bottom outlet.

At the same inlet flow rate, the oil phase medium exhibits a centripetal motion after separation in the cylindrical section, mainly distributed in the forced vortex zone at the center of the hydraulic cyclone. The oil phase medium flows towards the overflow port through the axis of the hydraulic cyclone. The closer it is to the overflow port, the higher the density of the oil phase distribution, and a small amount of oil phase medium flows out from the bottom flow port. As the inlet flow velocity increases, as shown in Fig. 8, the swept range of the forced vortex zone of the hydraulic cyclone increases, and the phase distribution density of the oil phase medium flowing out through the bottom outlet becomes lower and the distribution area at the bottom outlet becomes smaller. Taking into account the influence of inlet flow velocity on the oil volume fraction and separation efficiency of the bottom flow outlet, and avoiding increasing the number of repeated separations, it is recommended that the optimal range of inlet flow rate be 3.5 ~ 4.0 m^3^/min under this operating condition.

### Effect of oil content in mixed liquid on oil removal efficiency

When selecting a hydraulic cyclone structure of 4–8, a hydraulic cyclone model with an overflow diversion ratio of 0.2 was made, and the oil removal experiment of the hydraulic cyclone was completed under the condition of an oil content of 10%~30% After the hydraulic cyclone stabilizes (60 s), randomly take samples from the bottom outlet and test the oil content. The results are shown in Table 5.

**Table 5:** The effect of oil content in mixed liquid on the oil removal efficiency of a shaped hydraulic cyclone.

Number	Inlet flow m^3^/min	Oil content %	Oil volume ml	water volume ml	bottom outlet oil volume fraction %	separation efficiency %
1	1.0	10.0%	5.8	168	3.34%	66.6%
2	2.0	10.0%	4.8	175.5	2.66%	73.4%
3	3.0	10.0%	4.4	169	1.40%	86.0%
4	4.0	10.0%	8.7	168	0.60%	94.0%
5	1.0	20.0%	7.2	140	8.71%	56.5%
6	2.0	20.0%	7.5	163	7.56%	62.2%
7	3.0	20.0%	6.3	152	5.22%	73.9%
8	4.0	20.0%	7.4	157	2.54%	87.3%
9	1.0	30.0%	32.8	144.5	18.49%	38.3%
10	2.0	30.0%	18.3	115	13.72%	54.3%
11	3.0	30.0%	19.7	159	11.02%	63.2%
12	4.0	30.0%	15.8	190	7.67%	77.0%

When using a hydraulic cyclone with an overflow diversion ratio of 0.2 for oil-water separation, under the same oil content in the mixed liquid, such as when the oil content is 10%. As the inlet flow rate increases from 1.0 to 4.0 m^3^/min, the oil volume fraction at the bottom outlet of the hydraulic cyclone gradually decreases from 3.34% to 0.60%. The effective separation efficiency of oil-water mixture increased from 66.6% to 94.0%, showing an increasing trend. However, when the oil content of the mixed liquid increased to 30%, as the inlet flow rate increased from 1.0 to 4.0 m^3^/min, the oil volume fraction at the bottom outlet of the hydraulic cyclone gradually decreased from 18.49% to 7.67%. And the optimal oil-water separation efficiency of the mixed solution decreased from 94.0% to 77%. Under the same inlet flow rate, it is much higher than the case of an oil content of 10%. This indicates that the higher the oil content, the lower the oil-water separation efficiency of the hydraulic cyclone. This is mainly related to the viscous drag force of the oil content on the separation process of the mixed liquid, which affects the radial partial pressure and tangential flow velocity of the mixed liquid water phase medium.

**Fig. 10:**
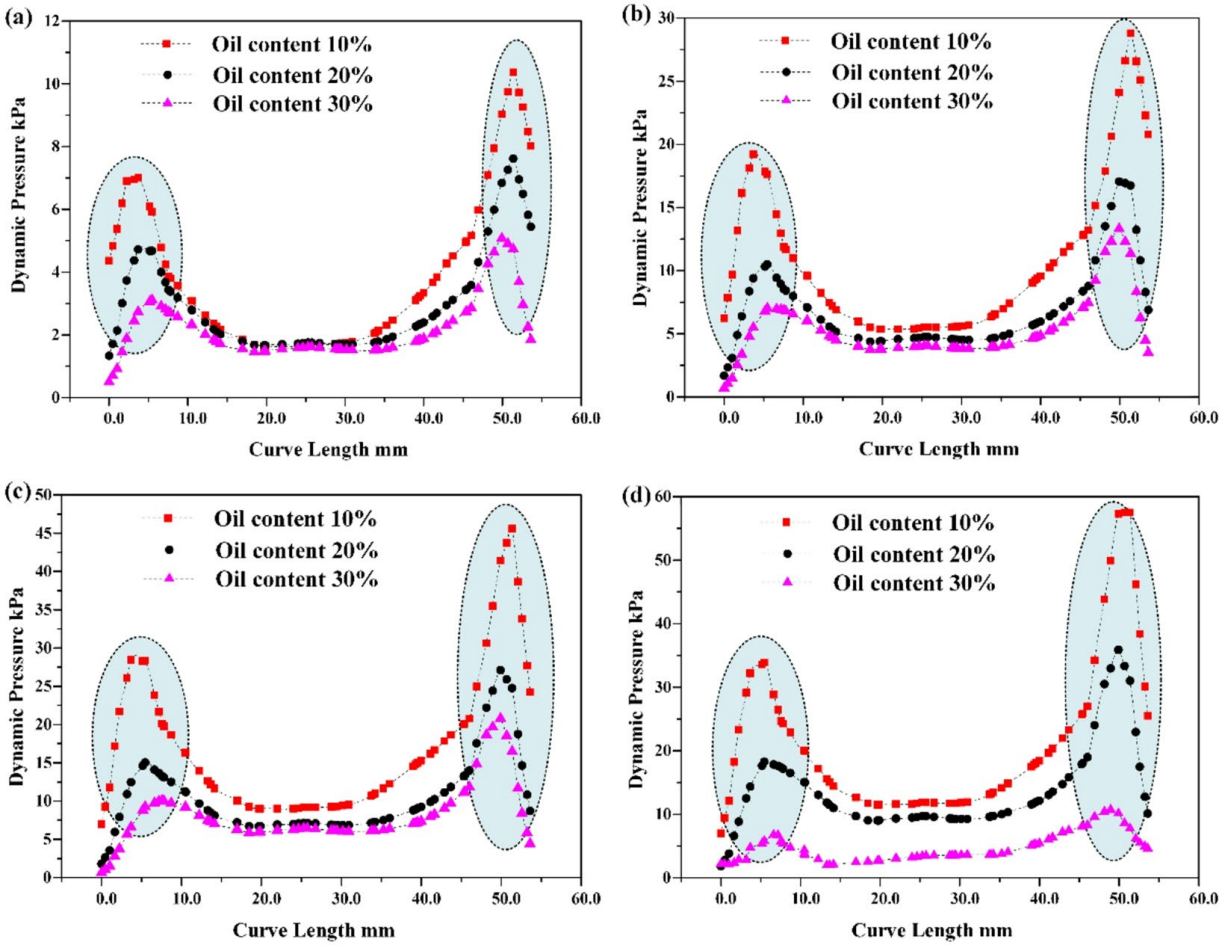
Radial distribution characteristics of hydraulic partial pressure in the cone section of the hydraulic cyclone

**Fig. 11:**
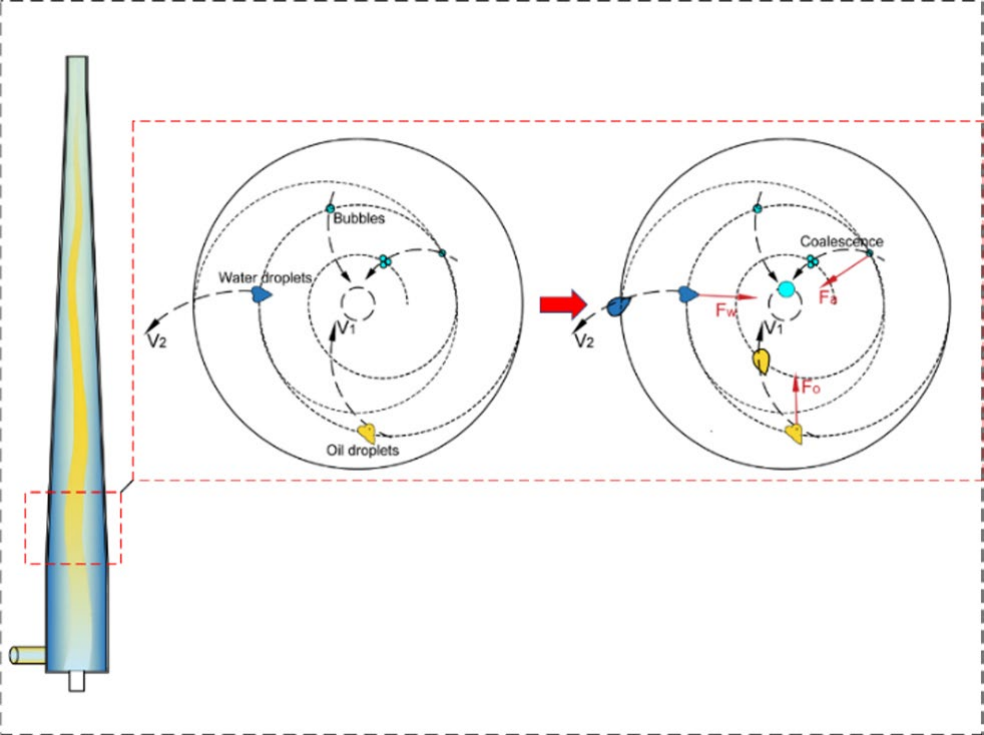
The effect of viscous drag and centrifugal force on the mixed liquid in the cone section of the cyclone

The radial distribution monitoring of hydraulic partial pressure in the cone section of the hydraulic cyclone shows that it is shown in Fig. 10. At the same inlet flow rate, as the oil content of the mixed liquid increases, the radial hydraulic partial pressure of the cone segment shows a decreasing trend. And the higher the oil content of the mixed liquid, the more obvious this trend becomes, mainly because the increase in oil content of the mixed liquid will lead to an increase in the concentration of viscous phase. When the oil phase medium and water phase medium of the mixed liquid flow at the same tangential velocity, the density difference causes the oil phase medium and gas phase medium in the mixed liquid to undergo centripetal motion while the water phase medium undergoes centrifugal motion. As shown in Fig. 11, coalescence of small-sized droplets occurs during this process. Whether it is the centripetal motion of oil and gas phase media or the centrifugal motion of water phase media, the coalesced droplets will be subjected to viscous drag force. Under the combined action of viscous drag and centrifugal force, the radial resistance experienced by the mixed liquid in the cylindrical and conical sections of the cyclone increases. The radial partial pressure of the aqueous medium decreases, and the separation efficiency also decreases accordingly.

**Fig. 12:**
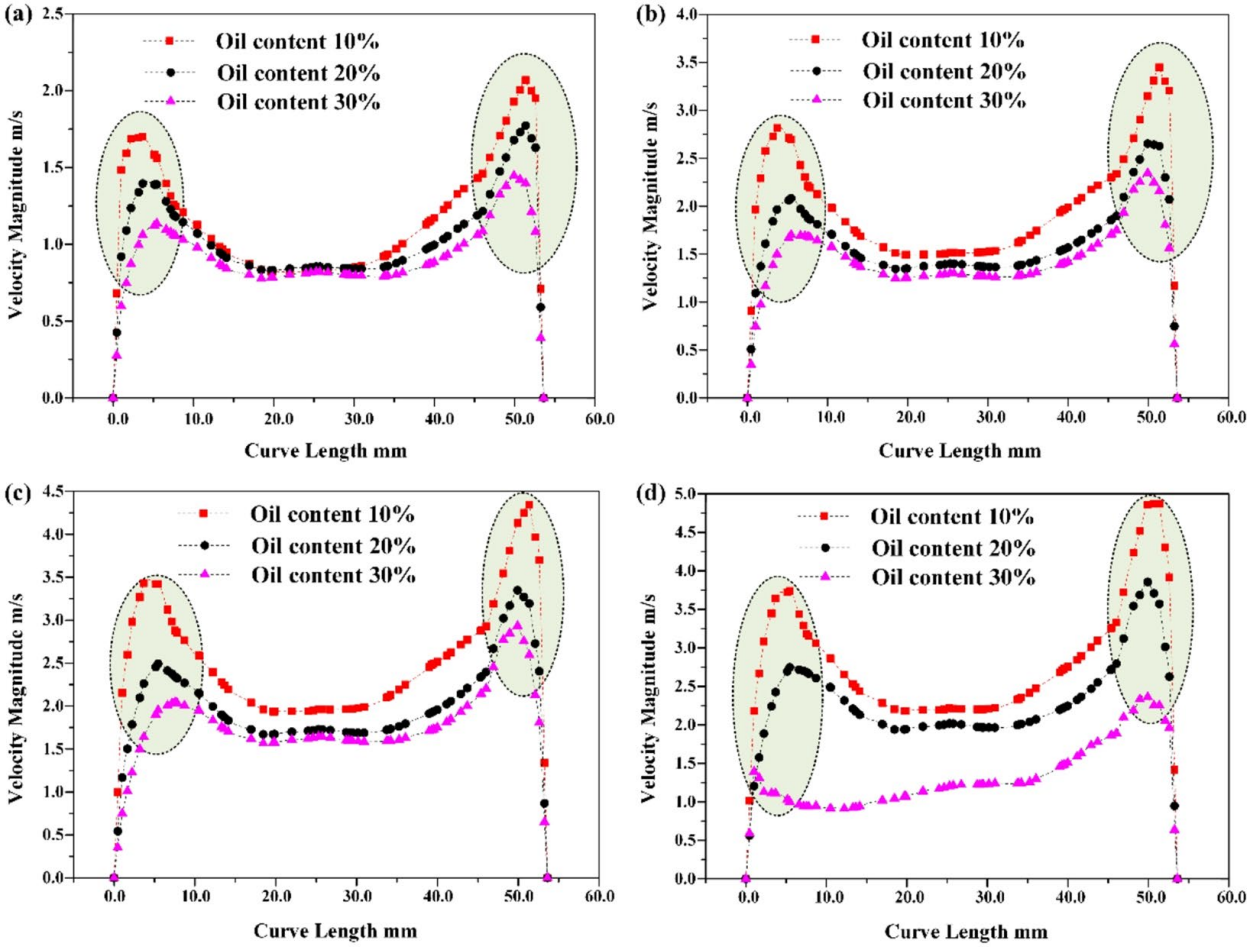
Distribution of tangential flow velocity in the cone section of the hydraulic cyclone

In addition, due to the viscous drag force acting on the mixed liquid in the cone section, the resistance along the way increases, and its tangential flow velocity also decreases. The monitoring results are shown in Fig. 12. The higher the oil content of the mixed liquid, the lower the radial partial pressure of the aqueous medium, but the higher the viscous drag force. The greater the radial resistance experienced during centrifugal motion, the lower the effective oil-water separation efficiency of the mixed liquid.

### Oil water separation efficiency

The effective separation efficiency of oil-water mixtures is an important parameter for evaluating the separation performance of hydrocyclones. The separation capacity of a hydraulic cyclone is usually calculated and evaluated based on its actual separation efficiency, as shown in Eq. (11). The numerical simulation process calculates the oil volume fraction at the bottom outlet by setting monitoring curves at Z = 0 and X = 0 of the hydraulic cyclone; During the experiment, the oil volume fraction at the bottom outlet was calculated based on the sampling results.

**Fig. 13:**
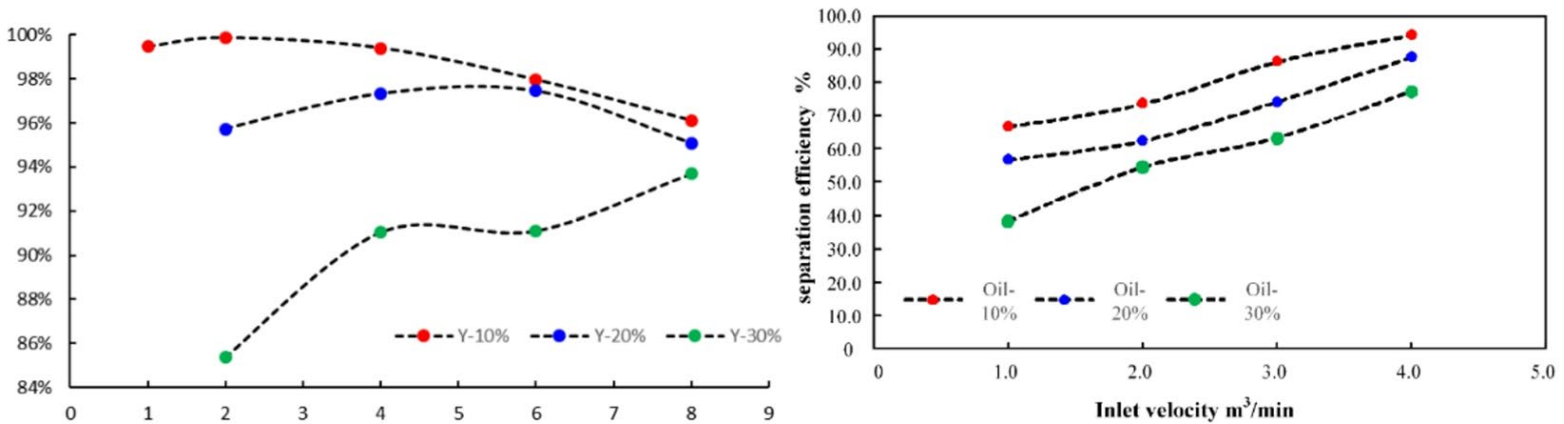
Efficiency of Mixed Liquid Hydraulic Cyclone Separation

The oil content and inlet flow rate of the mixed liquid have an impact on the oil removal efficiency; As shown in Fig. 13 (a), under experimental conditions, when the oil content of the mixed liquid is 10%, the oil removal efficiency of the hydraulic cyclone can reach 99.89%^26^. As the oil content of the mixed liquid increases, the oil removal efficiency of the hydraulic cyclone decreases, especially when the oil content of the mixed liquid reaches 30%, the maximum oil removal efficiency of the hydraulic cyclone is only 93.69%. Under simulated conditions, at different inlet flow rates, as the inlet flow rate increases, mixed liquids with different oil contents exhibit different characteristics, as shown in Fig. 13 (b) When the oil content is 10%, as the inlet flow rate increases from 1.0m^3^/h to 4.0m^3^/h, the oil removal efficiency at the bottom outlet increases from 69.79% to 93.47%; When the oil content is 20%, as the inlet flow rate increases from 1.0m^3^/h to 4.0m^3^/h, the oil removal efficiency at the bottom outlet increases from 57.21% to 87.63%; When the oil content of the mixture is higher than 20%, an increase in inlet flow rate will also increase the oil removal efficiency of the mixture, but it is still lower than the cases where the oil content of the mixture is 10% and 20%. In this type of hydraulic cyclone and under this operating condition, the overall trend is that the higher the oil content, the lower the oil-water separation efficiency, which is mainly related to the concentration of the dispersed phase in the mixed liquid^27–29^. An increase in oil content will lead to an increase in the concentration of the dispersed phase, while the average density of the mixed liquid will decrease, resulting in a decrease in the centrifugal force borne by the mixed liquid system. By increasing the inlet flow rate, the centrifugal rate of the mixed liquid hydraulic cyclone separation can be increased, thereby improving the separation efficiency.

## Conclusion

When using a standardized hydraulic cyclone for the treatment of oily wastewater, the overflow diversion ratio, inlet flow rate, and mixed liquid oil content of the hydraulic cyclone will all have an impact on the oil removal efficiency. In our experiments and numerical simulations, we obtained the following conclusions,

1. When the viscosity of the mixed liquid oil phase is 27–31 mPa·s, an increase in the overflow diversion ratio is beneficial for the rapid separation of low-density phase media. If the diversion ratio is too high, it will cause the liquid flowing out of the overflow port to form an oily mixture again. When the oil content of sewage is 10%, taking into account the changes in oil volume fraction at the bottom outlet, oil volume fraction at the overflow outlet, and oil removal efficiency, the optimal overflow diversion ratio of 0.2 is ultimately selected, and the effective oil removal efficiency can reach 92%.
2. The influence of inlet flow rate on the oil removal efficiency of the hydraulic cyclone is mainly reflected in the distribution of the mixed liquid phase. In the case of a constant oil content, an increase in inlet flow velocity will promote an increase in pressure gradient between the inner and outer vortices. During the inward migration of low-density oil phase media, coalescence occurs to form oil droplets, which experience an increase in centrifugal force in the forced vortex zone and a decrease in the oil volume fraction at the bottom outlet. To avoid increasing the number of repeated separations, it is recommended that the optimal range of inlet flow rate be 3.5 ~ 4.0 m^3^/min under this operating condition.
3. The influence of the oil content in the mixed liquid on the oil-water separation efficiency of the hydraulic cyclone is achieved through the interaction of viscous drag and centrifugal force on the radial partial pressure and tangential flow velocity. As the oil content of the mixed liquid increases, the radial flow resistance experienced by the cylindrical and conical sections of the hydraulic cyclone will increase, and the radial partial pressure, pressure gradient, and radial flow velocity of the aqueous medium will decrease, resulting in a decrease in separation efficiency.

## Data Availability

All data generated or analysed during this study are included in this published article.
